# Experiences of pain debut and healthcare received in men with chronic pelvic pain syndrome

**DOI:** 10.1186/s12894-023-01276-9

**Published:** 2023-06-13

**Authors:** Shirin Zarur, Louise Danielsson

**Affiliations:** 1 Institute of Neuroscience and Physiology, Department of Health and Rehabilitation, Sahlgrenska Academy, University of Gothenburg, Box 455, Gothenburg, 405 30 Sweden; 2 Friskare Fysik, Stora Nygatan 40, Malmö, 211 37 Sweden

**Keywords:** CPPS, Chronic prostatitis, Pain, Chronic primary pelvic pain

## Abstract

**Background:**

Chronic Pelvic Pain Syndrome (CPPS) is the occurrence of chronic pelvic pain when there is no proven infection or other obvious local pathology that may account for the pain. It is often associated with negative cognitive, behavioural, sexual or emotional consequences, as well as with symptoms of lower urinary tract, sexual or bowel dysfunction. As there is a close link between psychosocial factors and the development of myofascial pain syndromes it is important for healthcare professionals to have knowledge of how the pain begins and the activities at the debut of the symptoms.

**Aim:**

The aim of the study was to explore men’s experiences of the process leading to CPPS and healthcare received.

**Methods:**

Information was obtained from semi-structured video interviews with 14 men with CPPS. Interviews were audio-recorded and transcribed. The text was then abstracted into codes and analysed with inductive content analysis.

**Results:**

The age of the informants ranged between 22 and 73 (median 48), and the duration with CPPS ranged from 1 to 46 years. Two themes emerged, one with the heading *Struggling to pin it down* with four subthemes and *The helpful and unhelpful healthcare* with two subthemes. The four subthemes show that the informants experienced difficulties in their lives in the months before the debut of symptoms, for some it was several years. They had specific triggers for the onset of pain. These included cold, trauma to the perineum, chlamydia infection and possibly secondary to a symptomatic urethral stricture. Confusion and frustration were an important element in the informants’ overall experience of CPPS. Healthcare varied widely. The two subthemes about healthcare show expressions of being overlooked or wasting the doctor’s time, but also the experience of being validated and being thoroughly examined.

**Conclusion:**

The informants in our study described clear and specific triggers for CPPS such as being cold, having digestive issues and trauma to the perineum. Stressful events seemed to have a big impact on these informants and very possibly affected the start of symptoms. This information should be helpful healthcare professionals to understand the patient and his needs.

## Introduction

European Association of Urology updated the guidelines in 2022 on Chronic Pelvic Pain describing Chronic Primary Pelvic Pain Syndrome for men and women, previously called Chronic Pelvic Pain Syndrome (CPPS), as the occurrence of chronic pelvic pain when there is no proven infection or other obvious local pathology that may account for the pain [1]. However, CPPS is as of yet the internationally recognised term and will be used in this paper. To be called chronic, the pain must be continuous for three months or cyclic for at least six months. CPPS is often associated with negative cognitive, behavioural, sexual or emotional consequences, as well as with symptoms suggestive of lower urinary tract, sexual, bowel or gynaecological dysfunction. Several other primary pain syndromes associated with the pelvis are grouped under CPPS [1]. Muscular or myofascial origin is presented as one of the subclassifications for causes of pain [1–3]. A classification system for men and women based on symptoms has been proposed by Shoskes et al. [4, 5]. The mnemonic UPOINT consists of six domains that classifies the patients based on phenotypes; urinary symptoms, psychosocial dysfunction, organ specific findings, infection, neurologic/systemic, and tenderness of muscles [4, 5]. A seventh domain regarding sexual dysfunction resulting in UPOINT(S) has been proposed by Magri et al. [6]. EAU proposes further assessment be based on phenotype and UPOINT [1]. The global prevalence of CPPS in men varies between 2 and 10% [7], it affects men of all ages and demographic subgroups, although lower education, lower income and unemployment were associated with more severe symptoms [8]. Common painful areas are the penis (base, shaft, glans or urethra), perineum, rectum, suprapubic, testicles, groin and tailbone / glutes [9]. Symptoms associated with CPPS other than pain in the pelvic area are sexual dysfunctions [10, 11], voiding symptoms [10], pain or discomfort during and/or after sexual climax, pain or discomfort during urination [8] and psychiatric complaints [10].

The aetiology of CPPS is multifaceted. Klotz et al. [12] discuss that high psychosocial burden has been found in patients with CPPS. Like other chronic pain syndromes, CPPS could be seen as a biopsychosocial condition in which life events, personal history, coping strategies, and behaviour patterns, but also social situations and communities, influence pain sensations and emotional reactions. This stresses the importance of holistic diagnostic and therapeutic management of these patients [12]. Understanding the underlying psychological factors (including depression, anxiety, stress) is important for understanding CPPS in men [13], although the tendency for catastrophizing has been shown to be common [14], and neuroticism could possibly be a common factor [15]. Men with CPPS experience impairment in the mental and physical domains of general as well as condition-specific health-related quality of life [16]. Clinically, individuals who often experience more stress and anxiety have an increased tendency to develop triggerpoints (TP), which can lead to a myofascial pain syndrome [17]. Mental and emotional stress have been shown to increase electromyographic activity in TP, while adjacent muscles without TP remain electrically unchanged [18].

Magistro et al. [19] have suggested that treatment can be better targeted by starting from a clinical phenotype based on UPOINT(S). This avoids unnecessary use of antibiotics or anti-inflammatory drugs [19]. However, Engeler et al. [1] present and rate different treatment methods based on the primary pain symptom following the standard levels of evidence used by the EAU [1]. Manual treatment of pelvic myofascial TPs in these patients has shown to have a good outcome [20, 21] and is often used by physiotherapists. Cognitive Behavioral Therapy or other counselling has also had a positive outcome [20, 22]. A systematic review involving randomised controlled trials for non-pharmacological treatment concluded that acupuncture and shockwave therapy could provide pain relief in patients with CPPS [23]. A multimodal approach to treatment has been proposed [2, 19].

There are few qualitative studies on male CPPS [24–26]. An exploratory study found a link between pain and cold, where cold was involved in pain onset in four out of 10 informants, and eight subsequently associated cold with recurrent pain symptoms or worsening of ongoing pain. Heat was used by all informants to relieve pain [24]. Jonsson et al. [25] focused on the experience of living with male pelvic floor pain. There were several similarities with other chronic diagnoses stated by the informants, among other things, fatigue, impact on the workplace and relationships, and avoidance of social activities [25]. Wood et al. [26] present experiences of living with CPPS with a focus on the theme `Ongoing struggles for coping and cures: Obstacles and Aids´ which highlights, amongst other things, what men do to avoid triggering the pain, the search for cures and difficulties in talking about their illness [26]. There are yet no studies focused on the experience of the process and development of CPPS. As there is a close link between psychosocial factors and the development of myofascial pain syndromes it is important for healthcare professionals to have knowledge of how the pain begins, and the activities at the debut of the symptoms. The aim of the study was therefore to explore men’s experiences of the process leading into CPPS and healthcare received.

## Methods

### Design

A qualitative study was conducted using inductive content analysis that is described by Lindgren and Graneheim [27, 28]. The method is flexible for different types of data as it allows for different degrees of interpretation and layers of meaning in the data, which was assumed to suit the present study. The method is characterised by a focus on similarities and differences in the text and to analyse both the manifest and the latent content [27–29]. The researchers were inspired by a phenomenological approach, with the intention to understand the participants’ life-world and experiences of their lived bodies [30]. In this process, researchers’ pre-understanding needs to be held back and “bridled’’ [30] to open for new aspects to appear in the data and share the participants’ voices.

### Participants and recruitment

Recruitment of informants took place during spring and summer of 2022 via collegial networks within healthcare, including doctors at urology clinics as well as written information in waiting rooms at urology clinics announcements. Information was also posted for CPPS groups on Reddit and Facebook, and one group for strength training. Some informants participated due to a snowball effect. Most informants contacted SZ via email, and a few were contacted by SZ by telephone or via email after their therapist had disclosed their information at their request. We were aiming for informants in different age groups and levels of education, and all informants that wanted to participate were included. Similar aspects of content and meaning were noticed in the informants’ stories after eight to nine interviews, which indicated thematic saturation. Regardless, we wanted to interview a few more participants to avoid missing essential new data. The last few informants had a different type of pain onset, which was considered important for the variation of experiences. Men with continuous pain for three months or cyclic for at least six months, located in the pelvic region, or diagnosed with N50.8 F (Chronic Pelvic Pain Syndrome) or N41.1 (Chronic Prostatitis), were included. Exclusion criteria were pain from other genesis, for example, infection, neurological origin / disease with symptoms below the waist, previous or current cancer in the pelvic region, or not being able to hold a conversation in Swedish. The informants participating came from different parts of Sweden.

### Procedures and data collection

Individual interviews were conducted by SZ digitally on Zoom, via Gothenburg University’s licence with a password requirement from the participant which was sent via email. Interviews lasted between 24 and 67 min and were conducted during spring and summer of 2022. General demographic data was collected at the start of the interview. The semi structured interviews included the following topics: Information about diagnosis and start of symptoms, symptoms experienced, how and when it started, what life was like generally in that period, triggers for symptoms, feelings and thoughts regarding the pain, and how everyday life is at present time. The focus of the interviews was to gather information about the period when the symptoms started, and how it unfolded in the months/years after through the informants’ lived experiences. Open questions were used; “Can you tell me about when you first started feeling symptoms in your pelvis?”, “How was that period of your life in general?” and “Can you tell me about the symptoms you have/have had?”.

### Analysis

The interviews were audio-recorded and transcribed verbatim. Every interview was read several times by both authors. The analysis was a process in which the informants’ texts were divided into meaning units that were condensed. The text was then abstracted into codes and analysed with reflection and alternative interpretation for meaning. Both authors read all transcripts. The authors analysed and coded two interviews independently and then discussed their coding in detail. Thereafter, SZ coded the remaining transcripts. The codes were grouped into domains to get an overview of the material. The domains that emerged were for instance symptoms, factors regarding background, healthcare, experience and emotions about their illness, and the start of symptoms. Based on the similarities and differences of the codes, tentative subthemes were developed to illuminate the meaning of the participants’ experiences. This was an ongoing process during several meetings where the two authors discussed patterns of meaning in the codes and the narratives as a whole. The findings reflected not only for what was common in participants’ experiences, but also for individual variations. The manifest content was expressed on a descriptive level in the form of subthemes. The latent content, which is the text’s underlying message, was expressed on an interpretive level in the form of themes [27, 29]. Trustworthiness was ensured by an ongoing process of reflection and discussion between the researchers SZ and LD continuously through the analysis process.

## Results

Fifteen men were interviewed but only 14 were included in the study. One participant was excluded as the pain likely came from a neurological origin and he was referred to a urology clinic. Five of the informants expressed that they have not really received a diagnosis, the other informants received their diagnoses between one week after start of symptoms to 16 years. See Table 1 for Sociodemographic data.

**Table 1:** Sociodemographic data

Informants (n)		14
Age at interview, years, median (min-max)		48 (22–73)
	< 30 (n)	2
	30–59 (n)	9
	> 60 (n)	3
Level of education		
	High School (n)	2
	University/higher education (n)	12
Age* at debut of symptoms, years, median (min-max)		34 (18–59)
	< 25 (n)	3
	26–40 (n)	5
	> 40 (n)	5
Duration* of CPPS°, years, median (min-max)		6 (1–46)
	< 5 (n)	6
	6–10 (n)	3
	> 11 (n)	4
Time ^ till diagnosis (months)		0,25–192
	< 1 month (n)	2
	2–5 months (n)	2
	> 6 months (n)	5

*One informant could not recall when his CPPS symptoms started, only 13 of 14 informants included. °Chronic Pelvic Pain Syndrome. ^Five participants did not know if they had received a diagnosis

An important feature in the informants’ overall experiences of confusion and frustration was interpreted as a theme. The struggle to grasp what the problem was, why it started and what could be done, was clear to the authors. This experience was reflected in the central theme *Struggling to pin it down*, with its four subthemes; Stressful events in life matter, The symptoms and their start, Is it over for me? - being worried & frustrated, and There’s a chance - about finding one’s own way.

**Fig. 1:**
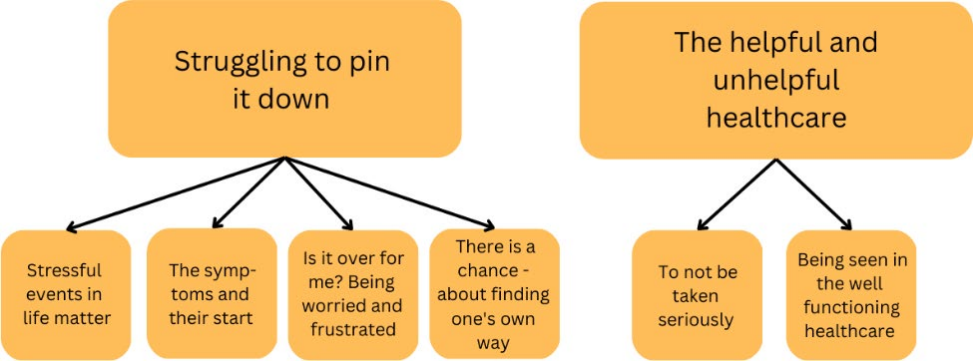
Themes and subthemes

A second theme emerged from the data; *The helpful and unhelpful healthcare* with the two subthemes To not be taken seriously and Being seen in the well-functioning healthcare. Some informants struggled to find adequate help from healthcare, others were taken care of immediately, and yet for some, the way to helpful care took a very long time. One informant never sought medical help at all. Figure 1 depicts the themes and subthemes.

### Struggling to pin it down

#### Stressful events in life matter

Most informants experienced distress during the months prior to the start of symptoms, for some of them it was years of complicated personal history. These events were described as stressful times at work with highly stressful workload, worrisome health issues, pressing personal lives, or multiple stressful events both in professional and personal life colliding. One informant described a violent family background, which led to a situation marked by threats from his mother and stepfather the months leading up to his marriage, which is when the CPPS symptoms started.

*“I grew up in a family where a lot of violence occurred between my parents. While talking to a Cognitive Behavioural Therapist during 4–5 months I remembered my mother used to hit me when I was little.” (Interview 6)*.

Stress at work was experienced by many informants, but also life-situations as a whole would cause stress;

*“You know life. You have to perform, you have to do something with your life, your job /…/ we have to row the whole package together.” (Interview 7)*.

*“I started a new job, worked far away, all week and slept there, and then had a long weekend at home. That job was pretty hectic, and it was a little hard at home. My partner and I both thought it was a little hard that we only met on the weekends, so I quit.” (Interview 8)*.

Experiences of several emotionally stressful events prior to the pelvic symptoms were described, for example, being in a bad relationship and simultaneously having a high load of stress, or the death of a parent, etc.;

*“Both my parents passed away, I’m an only child. During this chaotic time and the fear of having Multiple Sclerosis I also had an incredibly high workload. My wife’s career took off, so it was a perfect storm, I had to take more responsibility at home.” (Interview 5)*.

*“In my work as a journalist I’ve been exposed to threats, I’ve been persecuted and things like that, that was probably what caused post-traumatic stress during the Arab Spring.” (Interview 3)*.

One informant came to realise during the interview that around the same time as his CPPS symptoms started, his parents got divorced and he took on some extra responsibility for his teenaged little sister who was rebelling.

During these symptoms he thought he was just prone to being nervous. But the real disabling symptoms started at the age of 34 when there was increased stress at work, they had one small child and their second on the way;

*“I think I felt a little abandoned then. Because when a woman is pregnant, she goes into her bubble. And it was different from the first time when it’s completely new and unexplored and so on, so that time it felt like I, as a man, got to participate in a different way. But I feel like it wasn’t like that the second time.” (Interview 10)*.

Despite the fact that two informants did not have a stressful life situation leading up to the start of pain, stress still affected their symptoms. One informant experienced more somatic symptoms, while the other informant noticed that now, being retired from work, other things would cause stress that didn’t affect him previously.

#### The symptoms and their start

The symptoms described by the informants included pain or tenderness in the pelvic region, most often in the perineum, sometimes in the groin, suprapubic and lower back, the penis and the testicles, under the scrotum, or cramp in the perineum. In some cases it was more of a tension in the lumbar region or lower stomach. Urgency, weak urine stream, pain while urinating or emptying the bowels, and difficulty emptying the bladder were associated symptoms. One informant reported bladder dysfunction;

*“I can’t really feel; do I need to pee now or not, until it really feels like it is time, and I have to hurry to get to the toilet.” (Interview 5)*.

Intense urgency was the primary symptom in two cases, of which one had to take sleeping pills due to 24-hour symptoms. One informant reported that the symptoms mostly appeared at night, while during daytime he kept busy and didn’t really notice or think of it.

A few of the informants experienced pain during sexual orgasm or ejaculation, or pain after ejaculation when urinating, or discomfort during the erection. Other symptoms were pain while or after emptying the bowels, pain when emptying the bladder, or stiffness in the penis while flaccid.

A few of the informants could not recall exactly how the problems began as the symptoms started many years ago. For one informant, the symptoms slowly started during a vacation in London and for another informant, his previously mild symptoms got aggravated when his wife was pregnant with the second child. One informant suffered a trauma to the perineum;

*“I was standing on a railing on my boat and lost my grip and slid down with one leg on each side and hit the railing hard. 80 cm down and stop against a hard railing.” (Interview 13)*.

And yet another informant’s symptoms of CPPS seemingly started due to a symptomatic stricture in the urethra and painful surgeries to remove it. The rest of the informants also had specific but different triggers that started the symptoms. The triggers all have the pelvic region in common. Three stories were related to exposure to cold, for example, biking 10–12 h per week in the garage at wintertime, wearing wet swim shorts and swimming after a run when it was cold in the lake. Another trigger was medication for ADHD, and yet a different trigger was sexual intercourse, at ejaculation,

*“I collapsed from pain. My wife was completely terrified, and I was completely terrified. And then we were both terrified.” (Interview 6)*.

Another informant had a bad episode of lumbago, and his symptoms started after one month of being back at work sitting and driving a truck. Digestive issues were another trigger.

*“It started very sudden. It was a normal day, I had some trouble with my stomach that day, I was a little constipated. So I was on the toilet several times during the day. Then in the evening I sat on the toilet and I really strained to empty. After that I had a weird feeling in my stomach, in the pelvic floor, almost out in the penis. And the feeling did not give.” (Interview 5)*.

Two of the informants experienced the start of pain after a Chlamydia infection, even though it was treated successfully.

#### Is it over for me? - being worried and frustrated

The informants described that their tendency to feel worried and stressed seemed connected with their personality traits. They mentioned frequent worry, anxiety, being hard on themselves, being an emotional or sensitive person, holding back emotions, perfectionism and having a very strong sense of duty. Most informants had to deal with a lot of negative emotions tied to the symptoms and the process of finding answers. Hopelessness, frustration, catastrophizing and helplessness were expressed, especially when not finding information about their symptoms or treatments that would help. They described that there was very little information and guidance, which made them feel lonely. A lot of worry and fear was triggered in this process;

*“I thought it was cancer, something serious.” (Interview 6)*.

*“Worry took over everyday life.” (Interview 3)*.

Fear of making it worse by eating strong food, drinking alcohol and feeling limited in general was mentioned. Frustration and hopelessness came across as an important part of their story and experience of CPPS. One informant mentioned;

*“The thought: Is it over for me now? Am I an old man now? Is this the end? It’s a very difficult thought.” (Interview 4)*.

Discouragement when the medicine didn’t work or when they could not find a solution was described in different ways, for example;

*“When you notice that time goes on and you try different medications and so on but it doesn’t help, then it feels kind of hopeless. What is happening? Am I supposed to be like this forever?” (Interview 10)*.

Frustration of the uncertainty of when, where and how the pain started and persisted was also conveyed. Two informants expressed having had suicidal thoughts. Loneliness and shame was expressed by several informants as most of them did not have anyone to share these problems with and finding it a difficult subject to talk about, being a bit taboo.

Difficulty to talk about the symptoms also stemmed from the societal environment. Withdrawing from social life was a consequence expressed, and planning a social life was very difficult.

*“Didn’t want to do anything either. You couldn’t hang out with friends, just sat and thought about it all the time. This pain you have. Nothing would be fun to do anyway.” (Interview 11)*.

*“It’s stressful, you have to be on guard and kind of tense all the time, and it can affect how you behave and what you can do simply. Can’t get into anything, when it starts. One must constantly think; well, now it’s time, I have to get hold of a doctor here now, damn it’s Friday night, it’s not going to happen.” (Interview 9)*.

#### There is a chance - about finding one’s own way

When experiencing some relief of symptoms, the informants expressed that they regained hope of the condition getting better. They felt stronger and more motivated when, for example, having a pain free week and experiencing a normal life. Time also rendered some self-reflection and insight, which helped to understand oneself and factors that aggravated or alleviated the pain:

*“I will handle it if it gets very tense, because I know what to do to calm it down. I think that I might not ever completely get rid of it, but that I can live a normal life and maybe go months without pain, or years.” (Interview 1)*.

Having strategies for self-care and multiple tools to work with was mentioned. Strategies that gave a sense of control was important, and so was the understanding of connections;

*“I do the things I enjoy, instead of feelings of shame and anxiety. I think it has contributed to fewer relapses, or think less about it when I have relapses.” (Interview 2)*.

*“I have a wand (an S-shaped plastic wand used for internal trigger point treatments, authors remark) on the way, it’s easier to be dependent on myself. /…/ It’s almost like finding one’s own way and planning one’s own treatment. It’s so very individual how you react, what’s good for you and what isn’t.” (Interview 3)*.

Changes to behaviour and routines were made;

*“I gave up on the sexual ejaculation bit, which became a positive development. I enjoyed sexual intercourse without the need for ejaculation, enjoyment became focus.” (Interview 6)*.

Self-care factors that increase circulation were mentioned, examples include walks, meditation, exercises, stretching, swimming and sauna, alternating heat and cold, taking hot baths and massage. Mindfulness, healthy diet, talking with one’s family and spouse, physical activity, ejaculation, TENS (Transcutaneous electrical nerve stimulation) around the pelvic area and Percutaneous Nerve Stimulation were mentioned as helpful self-care treatments.

### The helpful and unhelpful healthcare

#### To not be taken seriously

Several informants had bad experiences with healthcare. They felt overlooked, or as wasting the doctor’s time. They narrated about very short meetings with the doctor, or being sent home from the emergency department without seeing a doctor, feeling worried of not being thoroughly examined;

*“He didn’t do a cystoscopy even though he mentioned it could be good to do that. He said he didn’t have time and he had to see many more patients.” (Interview 2)*.

Not being listened to was mentioned by the informant with the stricture in the urethra, as discussions regarding his CPPS with a doctor was for many years difficult. Receiving scarce information was also mentioned, for example, being handed a leaflet with general information about the syndrome.

When it took long to receive a diagnosis, the informants felt misled. For example, one informant was given treatment for constipation. Being unsure of the diagnosis was common and mentioned in different ways. A man who has had symptoms from the age of 34 stated that he was close to 50 when he saw a diagnosis in the journal. Others never really received a diagnosis.

*“Nobody said that it’s 100% this, the diagnosis was probably a combination of several people’s statements.” (Interview 5)*.

Some informants expressed that they only got short instructions and prescriptions of medications, and sometimes information that the condition was beyond hope of getting better;

*“The urologist made it worse for me, he basically said that this can’t be solved.” (Interview 1)*.

*“I went to the urologist and he just said; stress less, have lots of sex and do not eat strong food.” (Interview 7)*.

Yet another informant shared that the doctor had said, in a frustrated manner, that this comes and goes, and that he had to put up with it. Most of the informants were not informed about different professional perspectives and treatments, but had to find their own way in the system.

*“There’s like bulletproof glass between the urologists and the therapists, nobody mentioned or said anything about it.” (Interview 4)*.

#### Being seen in the well-functioning healthcare

The informants also reported positive healthcare experiences after being seen and confirmed in their health issues and symptoms, or being thoroughly examined and suggested potential treatments. One informant described a visit to the physiotherapist;

*“She examined from the feet upwards, she scanned my whole body. /…/ I am very stiff and tense, which I think has also affected me.” (Interview 1)*.

Some informants declared that they were notified of the diagnosis within a few days by their doctors, which they appreciated. Receiving confirmation about the condition was a relief; even though they knew that it was far from over, it felt calming to receive the diagnosis.

*“I was a little bit worried, but after the first examinations I wasn’t as worried at all. The general practitioner who had been at the urology department instilled confidence.” (Interview 5)*.

Having received a diagnosis made work life and sickness absence easier for one informant, who could then explain for his employer the reason to go to the healthcare centre. Another was never worried about his pain once the diagnosis was given and explained;

*“I got a clear picture, this is something with the musculature, so it’s not something to do with, for example, some type of cancer or something like that.” (Interview 13)*.

Being referred by the doctor to specialised healthcare personnel and treatments were expressed in positive terms;

“*We have a good physiotherapist who works with this kind of problem.” (Interview 3)*.

*“I get a lot of help when I go to the urotherapist. Then you can also ventilate your stuff. You could say they almost become psychologists. And sometimes I also get tips from them.” (Interview 10)*.

Informants expressed the importance of being thoroughly interviewed and treated with dignity, respect and empathy, and to have a good conversation with the urologist was very important;

*“One may not say masculine and feminine, but on one hand [with one of the doctors] tough and macho, and on the other hand with the female doctor and sexologist who were humble, and expressed: maybe you should try this.” (Interview 4)*.

*“The difference was partly that it was a conversation, that it was possible to actually have a conversation about all my symptoms and my history, /…/where the pelvic floor pain problem is part of the whole picture and that I had the opportunity to ask questions to the doctor. And also that she was so accommodating and responsive.” (Interview 14)*.

## Discussion

This study showed that several of the informants had experienced stressful events in their life prior to the start of symptoms. To the best of our knowledge, this finding has not been illuminated in previous qualitative research. Although these stressful events might not have been explained in this way before, several studies have shown a link between psychosomatic and myofascial aspects in CPPS [12–15]. Several informants in our study had noticed that psychosocial factors affected them and their symptoms. There might therefore be a need for a holistic approach and multimodal treatment including physiotherapy and psychotherapy for these patients, also suggested by other researchers [2, 3, 12, 13, 19, 20, 22]. A meta-analysis revealed that physiotherapy and / or cognitive behavioural therapy result in clinically meaningful improvement of symptoms when the aetiology is psychoneuromuscular rather than bacterial [31].

The start of symptoms proved to be very different in the informants’ stories; specific triggers, trauma to the perineum, secondary symptoms to a stricture in the urethra or Chlamydia infection. It is possible that these triggers could increase tension in the pelvic floor. It has been shown that dysuria, testicular pain and tenderness is quite common in Chlamydia infection in men [32]. Cold has shown to be a trigger in previous research, possibly inducing vasoconstriction in the prostate [24]. Thus, it is important for primary clinicians to ask the patient about such triggers, exploring possible links between specific events and symptoms, which may increase the patient’s awareness and understanding of aggravating factors.

The subjects of shame and tabu were brought up several times by the informants. It was difficult to speak about a sensitive and private area that could affect one’s image and manhood in association with a “masculine” illness (25–26). This can lead to keeping one’s problems to oneself and withdrawing from social life [16, 25], similar to other chronic illnesses [33, 34]. Even though our study did not focus primarily on the experience of pain, we found several similarities with patients’ experiences of other chronic conditions. The content of our subtheme *Is it over for me? - being worried & frustrated* could be comparable with findings in a meta-synthesis on chronic pain where Crowe et al. [35] present the meta-themes “Invisible but real” and “Unpredictability”, with categories like *keeping to oneself* and *lack of control*. Similar to this, our subtheme reflects the participants’ frustration and withdrawing from social activities. The findings in our subtheme *There is a chance - about finding one’s own way* connects to Crowe’s et al. [35] meta-theme “Keeping going”, which captures the struggle of keeping occupied and learning preventative strategies.

Loneliness, worry and frustration, which most informants expressed, have a negative impact on chronic pain and are similar to previous studies (25–26). It becomes clear from the narratives in this study that a pain free period due to self-care and/or treatment is imperative for the patient to feel in control of the situation, and to keep the motivation for progress.

**Fig. 2:**
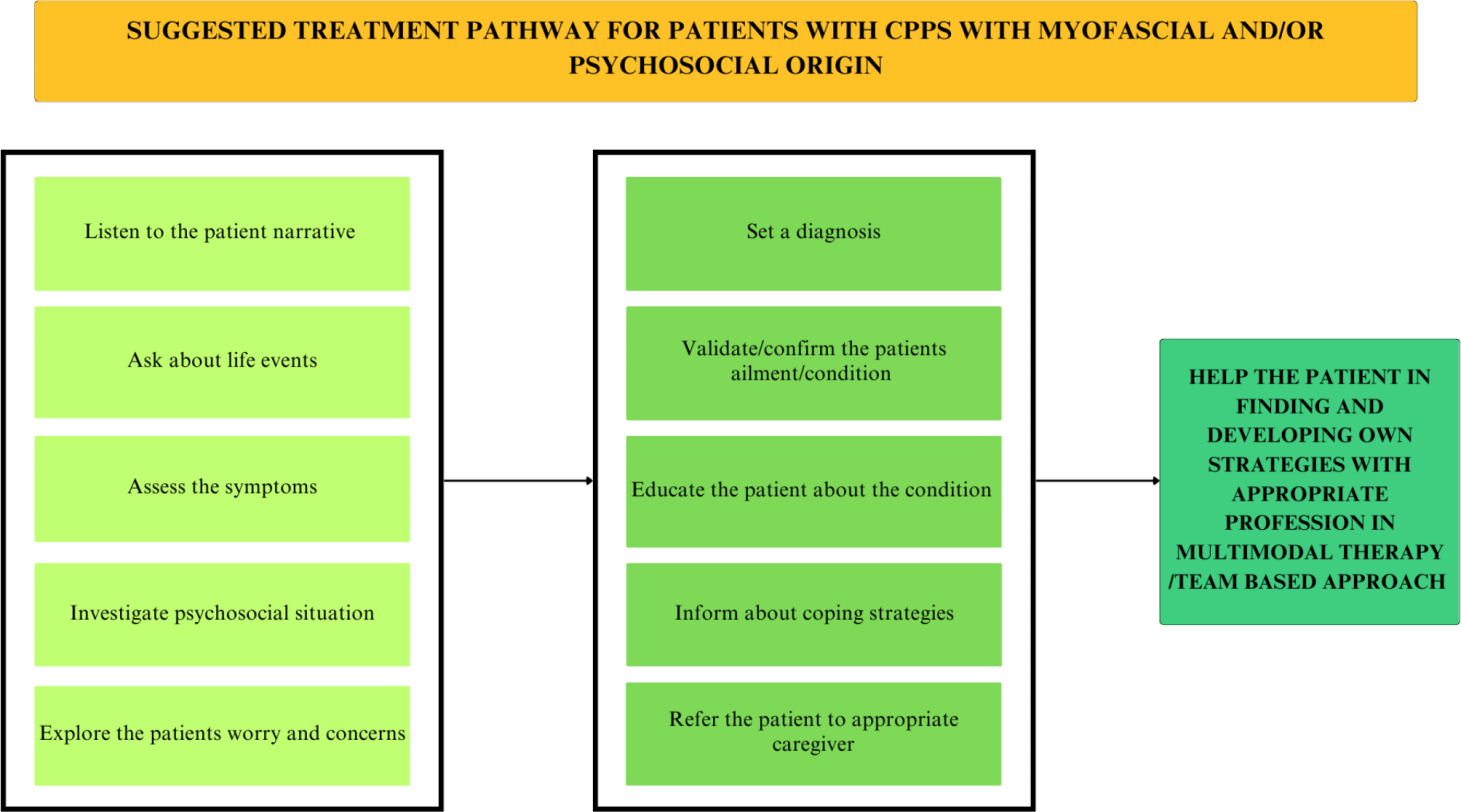
Suggested treatment pathway for Chronic Pelvic Pain Syndrome (CPPS)

Healthcare personnel have an important responsibility in their approach to worried patients. Several of the informants felt they were not seen properly, or taken seriously, which caused frustration and increased worry. The participants in our study emphasised the value of being seen and supported by the healthcare providers. The patient narrative is the first step in building partnership with the patient in Person-Centered Care (PCC) [36] and would be of value to understand and seek underlying causes to CPPS. This would be useful when informing the patient about the illness, to increase understanding of self-care and rehabilitation, and for adequate referral to healthcare professionals. If the right care is not provided, the healthcare system suffers financially as a result of incorrect efforts and time, while the patient’s suffering increases as tools to improve their pain are missing [37]. Much like other chronic illnesses, we suggest that PCC could be used in the care of men with CPPS, as it is advocated as a key component of effective illness management [36, 38, 39], and could be incorporated with the UPOINT(S) phenotype system. The results of this study showed that the patients felt calmer and better understood when they felt being listened to with openness and empathy. This suggests a need for doctors and other healthcare professionals to be responsive to the patient’s story and to be interested in their psycho-social situation. Based on the findings of our study, we suggest a model of important components in a treatment pathway, see Fig. 2 below.

Our results are likely primarily transferable to the CPPS population where myofascial origin is the primary cause of pain, nevertheless it might still be helpful to patients with other primary causes due to myofascial influence. More research is needed on CPPS as it is a complex illness and can be affected by both somatic and psychosomatic factors. This leads to questions about the most efficient treatment and best practice depending on causes of the symptoms and aggravating factors. The findings of stressful life events, muscular tension and chronic pain management support that physiotherapists with specialised knowledge of CPPS should be involved in the treatment. Manual treatment, relaxation, breathing exercises, grounding exercises, physical activity and stretching are a number of physiotherapeutic tools used in the clinic. A mixture of these could be used depending on the information shared in the interview and adapted depending on the outcome of the treatment. Treatment of patients with CPPS with myofascial influence could be managed most efficiently with a multimodal approach including physiotherapy and/or psychotherapy.

### Strength & limitations

To ensure scientific standard, Malterud [40] proposes that qualitative researchers consider these three criterias; reflexivity, transferability and interpretation/analysis [40]. Reflexivity concerns the researchers’ pre-existing knowledge and understanding, which in this case emerged from having worked with patients with CPPS (SZ). SZ had a notion that some patients might have been triggered into symptoms of CPPS and were affected by personal traits. To hold back the interviewer’s pre-conceptions, SZ aimed to keep a curious mind during the interviews to let the informants tell their stories and beliefs freely. Discussions with the second researcher (LD) were held continuously to critically reflect on the interviews and analysis as a way to handle subjectivity in the interpretation. Inductive analysis emerges from the relation between empirical data and theoretical conceptualisations [40]. In our case it stemmed from the grouping into domains concerning for instance symptoms, factors regarding background etc., and thereafter similarities and differences in regard to the aim and scope of the study, with the assumption that psychosomatic aspects might affect the start of CPPS. Quotes from several informants within each subtheme was used to exemplify and ground the interpretation in the data. The findings should be considered descriptions of how CPPS can unfold. However, there might still be other ways of how it could be triggered.

There was a wide span of both age and years with CPPS among the participating informants. This variation enables rich and nuanced data. However, it could be difficult for those informants that had decades of history with CPPS to remember the circumstances of when it started, with a risk of memory bias. Most informants reported a high level of education. Lower levels of education, income and employment are associated with more severe symptoms [8]. Therefore, we may not have captured their experiences with our sample, and the coping strategies mentioned might be difficult to implement for some groups. Overall, all the themes and subthemes were expressed in the 14 different narratives, except for one interview where the informant had mild symptoms. This informant did not share the frustration described by the other informants, as the symptoms did not affect his life to the same extent. We did not find any other patterns in the data that would suggest that certain characteristics among the informants predisposed them to experience a particular subtheme. However, the severity of symptoms and the healthcare professionals’ approach to these worried patients seem to have an impact on their progress. Possibly, the frequently reported stress, trauma to the perineum and genital infections may predispose patients to develop CPPS, which would be of interest to study in future quantitative investigations.

In our study, the interviewer was a woman interviewing participating men. Voluntary research participants have shown to adapt to the gender of the interviewer, modifying their responses to be more tactful [41]. Hence, the interviewer’s awareness of the potential threat to the masculine self was important, and the ability to respond in a sensitive way during the interview [42]. A lot of consideration went into making the informants feel comfortable and heard. Genuine interest was shown in each informant’s story using questions indicating that the informants are the experts in their situation. Before the interviews started, the informants were reminded of the voluntary nature of their participation and received an explanation of the aim of the study and how the interview would be guided [42]. Most informants commented that they were happy to share the information as they knew more knowledge was needed.

There are several benefits in using online interviews as they offer accessibility and flexibility, minimise time consumption and travel costs, while recruitment can expand beyond geographic barriers [43]. However, online interviews could negatively impact the interviewer’s ability to develop a sense of intimacy and trust, and potentially less rich interviews. Nonetheless, video interviews may replicate some of the opportunities of face-to-face interviews as non-verbal visual cues such as facial expressions, tears, gestures and other indicators of participant affect are present. This has been appreciated by informants in previous research to help them feel more connected to the researcher [43]. Technological issues can distract or disturb the interview, which was experienced very shortly in two interviews.

## Conclusion

Our study showed that the informants had experienced clear and specific triggers for CPPS such as being cold, having digestive issues and trauma to the perineum. Stressful events seemed to have a big impact on these informants and very possibly affected the start of symptoms. The participants’ experiences of frustration and hopelessness with their illness and not having been seen properly in the health care show the importance of asking about their emotions and experience of care. It is also important to explore factors that are relevant to the debut of pain. A person-centred approach with the possibility to receive multimodal treatment, for example including physiotherapy and/or psychotherapy could be beneficial, and needs further exploration in future research. This information should be helpful for health care professional to understand the patient and his needs.

## Data Availability

The datasets generated during the current study are not publicly available due to the sensitive nature of the data and the ability to identify participant’s identity from the raw data. The data (in Swedish) may be available from the corresponding author on reasonable request.

## List of abbreviations

EAU: European Association of Urology
CPPS: Chronic pelvic pain syndrome
UPOINT(S): Urinary symptoms, psychosocial dysfunction, organ specific findings, infection, neurologic/systemic, tenderness of muscles, sexual dysfunction
TP: Trigger point
PCC: Person-Centered Care.
